# ATPR triggers acute myeloid leukaemia cells differentiation and cycle arrest via the RARα/LDHB/ERK‐glycolysis signalling axis

**DOI:** 10.1111/jcmm.15353

**Published:** 2020-05-11

**Authors:** Yan Du, Mei‐ju Zhang, Lan‐lan Li, Xiao‐Lin Xu, Hao Chen, Yu‐bin Feng, Yan Li, Xiao‐qin Peng, Fei‐hu Chen

**Affiliations:** ^1^ School of Pharmacy Anhui Province Key Laboratory of Major Autoimmune Diseases Anhui Institute of Innovative Drugs Anhui Medical University Hefei China; ^2^ Institute for Liver Disease of Anhui Medical University Anhui Medical University Hefei China; ^3^ Department of Pharmacy the First Affiliated Hospital of Anhui Medical University Hefei China

**Keywords:** 4‐Amino‐2‐Trifluoromethyl‐Phenyl Retinate (ATPR), Acute myeloid leukaemia (AML), All‐trans retinoic acid (ATRA), Glycolysis, Lactate dehydrogenase B (LDHB), Raf/MEK/ERK signalling

## Abstract

Acute myeloid leukaemia (AML) remains a therapeutic challenge and improvements in chemotherapy are needed. 4‐Amino‐2‐trifluoromethyl‐phenyl retinate (ATPR), a novel all‐trans retinoic acid (ATRA) derivative designed and synthesized by our team, has been proven to show superior anticancer effect compared with ATRA on various cancers. However, its potential effect on AML remains largely unknown. Lactate dehydrogenase B (LDHB) is the key glycolytic enzyme that catalyses the interconversion between pyruvate and lactate. Currently, little is known about the role of LDHB in AML. In this study, we found that ATPR showed antileukaemic effects with RARα dependent in AML cells. LDHB was aberrantly overexpressed in human AML peripheral blood mononuclear cell (PBMC) and AML cell lines. A lentiviral vector expressing LDHB‐targeting shRNA was constructed to generate a stable AML cells with low expression of LDHB. The effect of LDHB knockdown on differentiation and cycle arrest of AML cells was assessed in vitro and vivo, including involvement of Raf/MEK/ERK signalling. Finally, these data suggested that ATPR showed antileukaemic effects by RARα/LDHB/ ERK‐glycolysis signalling axis. Further studies should focus on the underlying leukaemia‐promoting mechanisms and investigate LDHB as a therapeutic target.

**Highlights**

1. ATPR inhibits proliferation while promoting differentiation of AML cells.
2. Depletion of LDHB contributes to the growth of AML cells via the promotion of cell cycle arrest and blocking granulocytic differentiation in vitro and vivo.
3. Knockdown LDHB expression activates the Raf/MEK/ERK signalling pathway.
4. ATPR shows the antileukaemic effects by RARα/LDHB/ ERK‐glycolysis signalling axis.

## 1 INTRODUCTION

Acute myeloid leukaemia (AML) is a haematological malignancy characterized by abnormal proliferation of immature myeloid cells, with impaired differentiation and maturation.^1^ Despite progress in prevention, detection and treatment of AML, its recurrence and mortality rates remain high.^2^, ^3^ Therefore, this highlights that the development of differentiation therapy for leukaemia requires other highly effective and safe drugs. 4‐Amino‐2‐Trifluoromethyl‐Phenyl Retinate (ATPR), a derivative of all‐trans retinoic acid (ATRA), designed and synthesized by Anhui Medical University.^4^, ^5^ Our previous studies have shown that ATPR had a superior anticancer effects compared with ATRA on human gastric cancer,^4^ hepatocellular carcinoma,^6^ gastric carcinoma,^7^ breast cancer and leukaemia.^8^, ^9^, ^10^, ^11^ However, the molecular mechanism by which ATPR suppresses AML progression remains to be elucidated.^12^

While our understanding of cancer metabolism is still developing, altered metabolism is already recognized as a cornerstone mechanism of tumorigenesis.^13^ Glucose metabolic reprogramming from oxidative to aerobic glycolysis, refer as the Warburg effect, is a hallmark of cancer. This metabolic reselection contributes to multidrug resistance and is one of the reasons for the increase in cancer‐related mortality.^14^ Accumulating evidence suggests that glycolysis plays pivotal roles in tumour proliferation, metabolism, migration and invasion. Therefore, inhibition of glycolysis is a promising anti‐tumour strategy. Lactate dehydrogenase (LDH) is a key enzyme in glycolysis that catalyses the mutual conversion of lactate and pyruvate, NAD +, and NADH.^15^ LDH has two types of subunits: LDHA and LDHB, and the combination of the two subunits yields five kinds of tetramers in different proportions. LDHA is known to be elevated in a variety of tumour cells and plays an important role in tumour development and maintenance.^16^ However, compared with LDHA, the potential regulatory roles and molecular mechanisms by which LDHB affects the development and progression of AML remain largely unknown.

Raf/MEK/ERK signal pathway, also known as ERK signalling pathway, is composed mainly of a three‐stage enzyme‐linked functional unit, namely Raf, MEK and ERK excitation.^17^ The duration of ERK phosphorylation and activation is closely related to cell fate. Generally, continuous and appropriate activation can promote cell proliferation by promoting protein synthesis and improving protein stability. However, over‐activation of the ERK pathway can block the process of cell cycle. Recent studies have reported that PD98059 could block the activation of ERK1/2 and reduce the growth and differentiation of AML cell lines induced by dodecyl gallate acid and gifitinib.^18^ U0126 significantly blocked the differentiation of human AML cell lines induced by LukS‐PV and pulsatilla saponin A via inhibiting the activation of ERK pathway.^19^ Abnormal expression of the Raf/MRK/ERK signalling pathway is closely associated with the development and malignant progression of a variety of malignancies and has been identified as a novel target in AML therapy.

Therefore, we hypothesize that LDHB is involved in AML progression via regulating cell metabolism pathways and investigate the underlying mechanisms by which ATPR show the antileukaemic effects via the RARα/LDHB/ERK‐glycolysis signalling axis. Furthermore, ATPR may have potential as a chemotherapeutic agent, and LDHB may act as a therapeutic target.

## 2 MATERIALS AND METHODS

### 2.1 AML patient samples and ethics statement.

Patients with newly diagnosed AML (n = 15) were recruited from the First Affiliated Hospital of Anhui Medical University. Peripheral blood was collected from patients and mononuclear cells were isolated by standard Ficoll‐Hypaque density centrifugation. Cells were washed with RPMI 1640 and subjected to various assays.

### 2.2 Materials

ATPR was synthetized by the School of Pharmacy, Anhui Medical University (Anhui, China). ATRA and Ro41‐5253 were purchased from Sigma‐Aldrich (St Louis, MO, USA). Both ATPR and ATRA were prepared as 10^‐2^ M dehydrated ethanol reserve solution and maintained at −20°C.

### 2.3 Cell culture

The AML cell lines NB4 and HL‐60 were purchased from Shanghai Genechem Co., Ltd. (Shanghai, China) and the KG‐1, U937 and MOLM‐13 cells were donated by the University of Maryland School of Medicine. The cells were maintained in suspension in RPMI‐1640 medium (Hyclone) containing 10% FBS (Biological Industries) in a humidified atmosphere of 5% CO_2_ at 37°C.

### 2.4 Stable cell line establishment assay

To generate stable LDHB knockdown cell populations, AML cells were infected with pHBLV‐U6‐MCS‐PGK‐PURO‐shLDHB or pHBLV‐U6‐MCS‐PGK‐PURO‐Scramble. Puromycin (5 mg/ml) was used to select for stably transfected cells for 2 weeks. The human LDHB shRNA sequence was designed as follows: 5′‐GGATATACCAACTGGGCTA‐3′. The control shRNA sequence was as follows: 5′‐TTCTCCGAACGTGTCACGTAA −3′. LDHB knockdown in the stable cell line was verified by Western blotting assay.

### 2.5 Cell viability assay

Cell Counting Kit‐8 (CCK‐8) assay was performed to test the cell viability. The cells were seeded in 96‐well plates with a density of 5000 cells per well and treated at different times (24h, 48h, 72h) and different concentration of ATPR (1*10^‐9^, 1*10^−8^, 1*10^−7^, 1*10^−6^, 1*10^−5^M). CCK‐8 (10 μl per well) solution was transferred to each well. After incubation for 4 h, the optical densities (ODs) were checked at 450nm using absorbance microplate reader (Bio‐Tek, ELX800). The inhibition rate calculated by the following formula: (1‐(OD450 of control‐OD450 of drug)/OD450 of control) ×100%.

### 2.6 Quantitative real‐time PCR (qRT‐PCR)

The total RNA of NB4 cells extracted with TRIzol reagent (Invitrogen Corp., Carlsbad, CA, USA), and the first‐strand cDNA was synthesized using a Thermoscript RT‐PCR synthesis kit (Fermentas, Canada) according to the manufacturer's instructions. Gene expression was determined using cDNA SYBR‐Green real‐time PCR Master Mix by quantitative real‐time PCR (Takara). The mRNA ratio of the target gene to β‐actin was calculated by using the 2^−ΔΔCt^ formula. The experiments were performed at least three times with three different templates. The primers used were:

β‐actin (forward: 5′‐CCCATCTATGAGGGTTACGC‐3′;

reverse: 5′‐TTTAATG TCACGCACGATTTC‐3′);

LDHB (forward: 5′‐CCTCAGATCGTCAAGTACAGTCC −3′

reverse: 5′‐ATCACGCGGTGTTTGGGTAAT‐3′);

RARα (forward: 5′‐AAGCCCGAGTGCTCTGAGA‐3′

reverse:5′‐TTCGTAGTGTATTTGCCCAGC‐3′);

RARβ (forward: 5′‐AAACGTCTGCCTGGTTTCAC‐3′

reverse:5′‐AAGGCCGTCTGAGAAAGTCA‐3′);

RARγ (forward: 5′‐TTCAGTGAGCTGGCTACCAA‐3′

reverse:5′‐CTTGTGCAGATACGCAGCAT −3′);

CRABP2(forward: 5′‐ ATCGGAAAACTTCGAGGAATTGC −3′

reverse:5′‐ AGGCTCTTACAGGGCCTCC −3′);

CYP26A1(forward: 5′‐CTGGACATGCAGGCATAAA −3′

reverse:5′‐GCCCCAGGTAAGTGATCAGA −3′);

LDHA (forward: 5′‐ATGGCAACTCTAAAGGATCAGC −3′

reverse:5′‐CCAACCCCAACAACTGTAATCT −3′);

HK2 (forward: 5′‐GAGCCACCACTCACCCTACT‐3′

reverse:5′‐CCAGGCATTCGGCAATGTG‐3′);

ENO1 (forward: 5′‐GCCGTGAACGAGAAGTCCTG‐3′

reverse:5′‐ACGCCTGAAGAGACTCGGT‐3′);

GAPDH (forward: 5′‐GGAGCGAGATCCCTCCAAAAT‐3′

reverse:5′‐GGCTGTTGTCATACTTCTCATGG‐3′);

### 2.7 Cell differentiation analysis

Cell maturation was evaluated by cellular morphology and the content of cell surface differentiation‐related antigen CD11b and CD14. Morphology was determined with the Wright‐Giemsa staining, and the content of CD11b and CD14 was acquired by CytoFLEX (Becton Dickinson, USA).

### 2.8 Cell cycle analysis

To analyse the intracellular DNA content, the cells were harvested and washed in cold PBS, and then they were fixed in 75% ethanol/25% PBS at −20°C overnight. A portion of the fixed cell suspension containing 1 × 10^6^ cells was washed twice in cold PBS. After then, cells were stained with 500ul of propidium iodide (PI) staining buffer (Beyotime, China), which contains 20μl RNase A, at room temperature for 30min in the dark. The cells were subjected to cell cycle analysis using CytoFLEX (Becton Dickinson, USA), and Modfit software was used to estimate G0/G1/S/G2/M phases of the cell cycle.

### 2.9 Western blotting analysis

Cultured cells were lysed with RIPA lysis buffer for 30 min for Western blotting (Beyotime, China). Then, it was centrifuged at 12,000 rpm for 40 min, and the supernatants were collected. Protein concentration was measured using a BCA protein assay kit (Boster, China). The whole‐cell extracts (20 mg of protein) were separated on 8%‐12% sodium dodecyl sulphate‐polyacrylamide gel electrophoresis (SDS‐PAGE) and blotted onto PVDF membranes (Millipore, Billerica, MA, USA). After blocking with 5% nonfat milk in TBST (3h), nitrocellulose blots were incubated overnight with appropriate primary antibodies (Beyotime, China). The protein blots were washed four times in TBST before incubation for 1 h in goat anti‐mouse or anti‐rabbit horse radish peroxidase conjugate antibody at 1:10 000 dilutions in TBST containing 5% skim milk. After washing four times with TBST, the protein blots were detected with ECL‐chemiluminescent kit (ECLplus, Thermo Scientific). Antibodies against human PU.1, Cyclin A2, Cyclin D3, CDK4, p‐MEK, MEK, Raf, p‐ERK 1/2, ERK 1/2 and LDHB (Abcam) were diluted at 1:2000 dilution and anti‐β‐actin (ZSGB‐BIO, China) was used at 1:300. Quantitative densitometric analyses of immunoblotting images were performed using Image J software.

### 2.10 Ki‐67 analysis

The AML cells were plated in 6‐well plate containing 2 mL RPMI‐1640 medium. The plated cells were treated with ATPR (10^‐6^M) and incubated for different times (24 h, 48 h, 72 h). Following this, cells were washed twice with PBS and fixed using fix solution for 60 min at 4°C. Fixed cells were spun at 1000g for 10 min and washed with PBS twice. Cells were resuspended in 1 mL of permeabilization buffer and incubated for 10min at room temperature. Cells were incubated for 12 h with primary antibody dilution buffer containing a polyclonal antibody to human Ki‐67 (1:400 dilution) at 4°C, and then with secondary antibody (1 h). Successively, cells were resuspended in 50 μL of DAPI staining solution (Beyotime) and observed under Fluorescence Inversion Microscope System (OLYMPUS).

### 2.11 CFDA‐SE cell proliferation assay

Cell proliferation determination was conducted by CFDA‐SE probe (Beyotime, China). Briefly, synchronized AML cells were harvested and washed three times with PBS. Afterwards, the cells were stained with CFDA‐SE in 6‐well plates according to the manufacturer's protocol. Furthermore, the cells were treated with ATPR (1*10^‐6^M), and incubated for different times (24 h, 48 h, 72 h). These analyses were performed on the flow cytometer CytoFLEX (Becton Dickinson, USA).

### 2.12 Tumour xenograft

Six‐week‐old female NCG mice were obtained from the Nanjing model animal research institute (Nanjing, China) and housed under pathogen‐free conditions in SPF animal house of Anhui Medical University (Hefei, China). Then, 100 μl of NB4 (LDHB shRNA/control shRNA) cells suspension containing 5 × 10^6^ cells was diluted with Matrigel and PBS (1:1). The cells were subcutaneously injected into the right flank of each mouse. Tumour sizes and mice weight were measured every day. Two weeks later, the mice were killed, and the tumours were removed for further assessment.

### 2.13 Histopathology

Tumour tissues were fixed with 4% paraformaldehyde (24 h), and embedded in paraffin blocks for routine histology. Haematoxylin and eosin (H&E) and immunohistochemistry (IHC) staining were performed according to a standard procedure. Human monoclonal LDHB, KI67 and CD11b antibody (Bioss, China) for IHC were used at a 1:300 dilution. The pathological changes were assessed and photographed under Fluorescence Inversion Microscope System (OLYMPUS).

### 2.14 Nitroblue tetrazolium (NBT) reduction assay

For the NBT reduction assay, cells were inoculated in a 6‐well plate and treated with different time of ATPR (1*10^‐6^M). A 10 μl aliquot of NBT solution, composed of 10 mg/ml NBT (Sigma‐Aldrich) and 2 µg/ml PMA(Sigma‐Aldrich), was added to each well, and then cells were incubated at 37°C for 30 min, then the proportion of cells containing the precipitated formazan particles in 300 cells was counted by optical microscope.

### 2.15 Glucose consumption, lactate production and ATP generation assay

The expression of glucose consumption, lactate production and ATP generation in supernatants of AML cells were evaluated using commercial assay kit (Glucose assay kit (F006‐1), Lactate colorimetric assay kit II (A019‐2), ATP colorimetric assay kit (A095‐1), Jiancheng Bioengineering Institute, Nanjing) according to the manufacture's protocol.

### 2.16 Oxygen consumption rate (OCR) assay

Oxygen consumption rate (OCR) was real time determined using Seahorse Bioscience XF extracellular flux analyzer (Seahorse Bioscience, USA) and experiments were detected according to the manufacturer's protocol.

### 2.17 Statistical analysis

Experimental data were presented as mean ± SD unless otherwise stated. Statistical significances determined by one‐way ANOVA, Student's t test and Bonferroni's test. *P* < 0.05 was considered to be statistically significant.

## 3 RESULTS

### 3.1 AML cell lines were sensitive to ATPR in a concentration‐dependent

**Figure 1:**
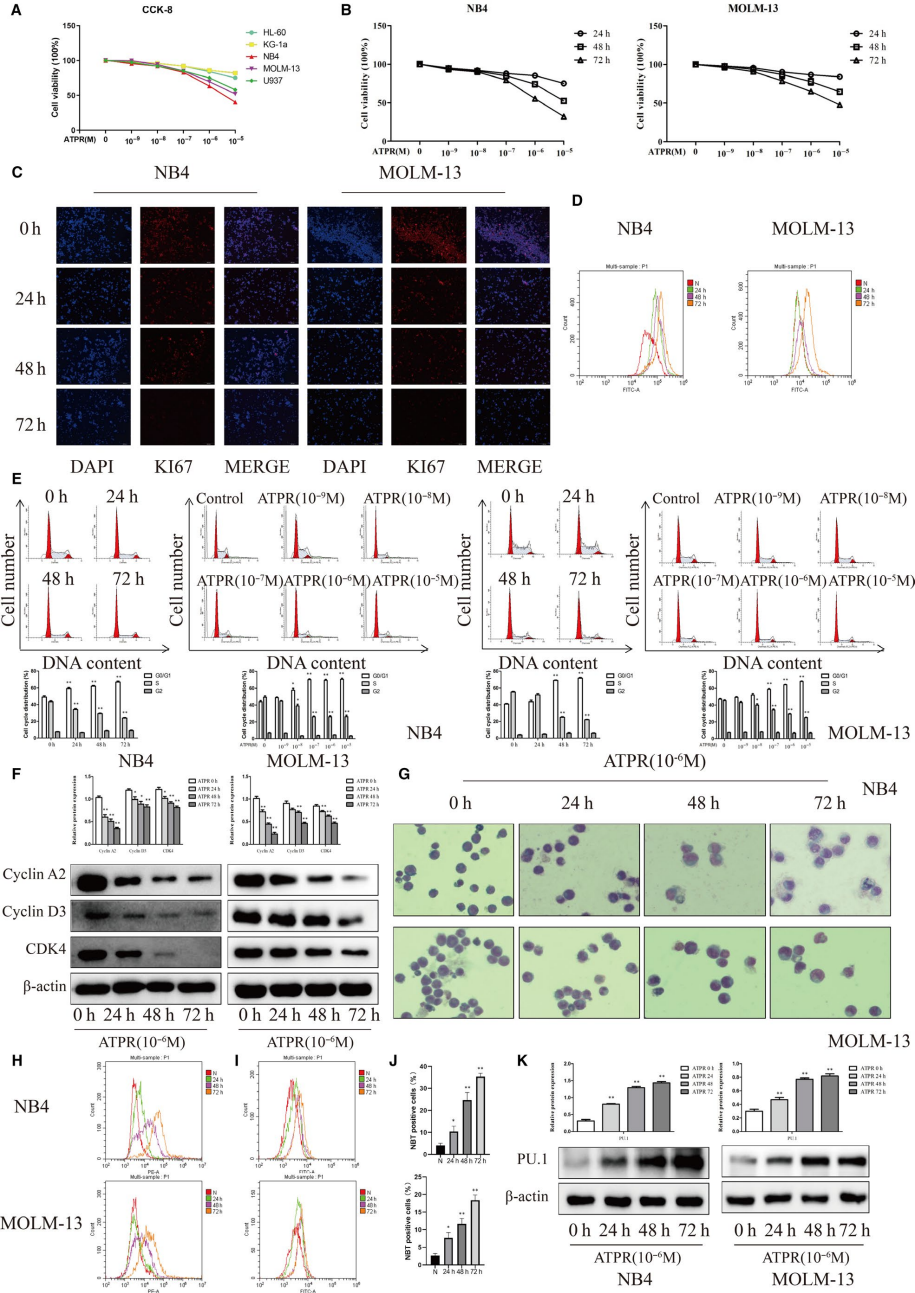
ATPR inhibits cells proliferation and differentiation of AML cells. (A) AML cells were exposed to various concentrations (0, 10^−9^, 10^−8^, 10^−7^, 10^−6^, or 10^−5^ M) of ATPR for 72 h, followed by the determination of cell viability using the CCK8 assay. (B) NB4 and MOLM‐13 cells were exposed to various concentrations (0, 10^−9^, 10^−8^, 10^−7^, 10^−6^, or 10^−5^ M) of ATPR for 24–72 h, followed by the determination of cell viability using the CCK8 assay. (C) NB4 and MOLM‐13 cells were treated with ATPR (10^−6^ M) for different durations (24–72 h). Immunofluorescence was used to detect KI67. (D) CFDA‐SE levels were assessed by flow cytometry. (E) NB4 and MOLM‐13 cells were treated with 0, 10^−9^, 10^−8^, 10^−7^, 10^−6^, or 10^−5^ M) of ATPR for 24–72 h. The cell cycle distribution was analysed by flow cytometry (Mean ± SD, n = 3). (F) NB4 and MOLM‐13 cells were treated with ATPR (10^−6^ M) for different durations (24–72 h). The protein expression of Cyclin A2, CDK4 and Cyclin D3 were assessed by Western blotting (Mean ± SD, n = 3). (G) NB4 and MOLM‐13 cells were stained with Wright‐Giemsa dye and the cell morphological features were observed under a microscopy. (H) CD11b expression was analysed by flow cytometer. (I) CD14 expression was analysed by flow cytometer. (J) NBT reduction experiment was performed to count the positive cell rate (Mean ± SD, n = 3). (K) The protein expression of PU.1 was determined by Western blotting (Mean ± SD, n = 3). *P < 0.05, **P < 0.01 versus control group

To investigate whether AML cells (NB4, HL‐60, KG‐1, U937 and MOLM‐13) were sensitive to ATPR treatment, the CCK8 assay was used. As shown in Figure 1A a markedly reduced viability and proliferation at concentrations of 10^‐7^ ~ 10^‐5^M were seen in 3/5 AML cell lines under ATPR treatment, indicating ATPR sensitivity in a subset of malignant tumour‐derived AML cells. According to the data, IC 50 of ATPR is HL‐60 (2.3∗10^−4^mol/L), KG‐1a (1.4∗10^−3^mol/L), NB4 (4.5∗10^−6^mol/L), MOLM‐13 (8.8∗10^−6^mol/L), U937 (2.1∗10^−5^mol/L). Moreover, the NB4 and MOLM‐13 were the most sensitive cells to ATPR treatment.

### 3.2 ATPR inhibited proliferation of AML cells

To profile the effects of ATPR on the proliferation of AML cells, we chose NB4 and MOLM‐13 cells to conduct the following experiment. As shown in Figure 1B, the growth curves of NB4 and MOLM‐13 cells treated with ATPR showed a time‐ and concentration‐dependent inhibition. A significantly decreased proliferation was determined by observing the expression of the proliferation antigen, Ki‐67 antigen, after ATPR treatment in a time‐dependent (Figure 1C). The CFDA‐SE dye showed that the cells treated with ATPR significantly decreased the average number of cells in CFDA‐SE profiles for 1 division after 48h and 72h as compared to 0h and 24h (Figure 1D). Flow cytometry showed that a time‐ and concentration‐dependent accumulation of cells in G0/G1 phase was observed after ATPR treatment. However, the percentage of cells in S phase was reduced (Figure 1E). As shown in Figure 1F, the marker proteins of G0/G1 phase (Cyclin D3, Cyclin A2, CDK4) of cells were arrested in the NB4 and MOLM‐13 cells treated with ATPR in a time‐dependent manner. These results indicated that ATPR inhibited proliferation of AML cells by arresting cell cycle at G0/G1 phase.

### 3.3 ATPR‐induced differentiation of AML cells

To profile the effects of ATPR on the cell differentiation of AML cells, cell maturation was evaluated by cellular morphology and the content of CD11b. Results of Wright‐Giemsa staining showed that an obviously kidney‐shaped shrinkage was shown in both NB4 and MOLM‐13 cells after ATPR treatment in comparison with negative control, representing that cells obtained the trend of differentiation (Figure 1G). Moreover, flow cytometry showed that ATPR remarkably increased CD11b and CD14 (granulocytic differentiation marker) expression levels in a time‐dependent manner (Figure 1H, I). Then, the NBT‐positive cells were increased (Figure 1J) and the protein levels of PU.1 were also remarkable elevated after ATPR treatment in a time‐dependent manner (Figure 1K). Altogether, these results indicated that ATPR inhibited proliferation and induced differentiation in AML cells with a time‐ and concentration‐dependent manner. The optimum anti‐tumour effect of ATPR for AML cells was 10^‐6^M concentration for 72 h.

### 3.4 ATPR induced the expression of target genes in AML cells containing a functional PML‐RARα

**Figure 2:**
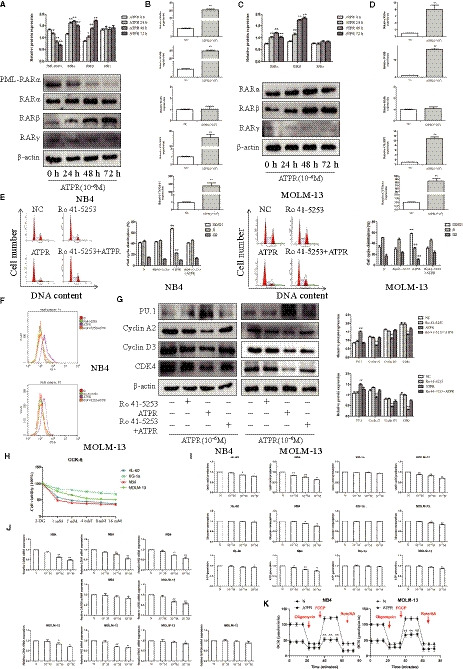
ATPR shows antileukaemic effects with RARα dependent. (A) The NB4 cells were treated with ATPR (10^−6^M) for different durations (24h–72 h). The protein expression of PML‐RARα, RARα, RARβ and RARγ was assessed by Western blotting (Mean ± SD, n = 3). (B) Values are presented as the mean ± SD (n = 3) of three in Quantitative real‐time PCR analysis of mRNA expression of RARα, RARβ, RARγ, CRABP2 and CYP26A1 treated with ATPR (10^−6^M) for 48h in NB4 cells‐dependent experiments. (C) The MOLM‐13 cells were treated with ATPR (10^−6^M) for different durations (24h–72h). The protein expression of RARα, RARβ and RARγ was assessed by Western blotting (Mean ± SD, n = 3). (D) Values are presented as the mean ± SD of three in Quantitative real‐time PCR analysis of mRNA expression of RARα, RARβ, RARγ, CRABP2 and CYP26A1 treated with ATPR (10^−6^M) for 48h in MOLM‐13 cells‐dependent experiments. (E) The cell cycle distribution was analysed by flow cytometry (Mean ± SD, n = 3). (F) CD11b expression was analysed by flow cytometer. (G) The protein expression of PU.1, Cyclin A2, CDK4 and Cyclin D3 was assessed by Western blotting (Mean ± SD, n = 3). (H) AML cells were exposed to various concentrations (0, 2, 4, 8 or 10mM) of 2‐DG for 72 h, followed by the determination of cell viability using the CCK8 assay. (I) AML cells were exposed to various concentrations (0, 10^−7^, 10^−6^, or 10^−5^ M) of ATPR for 72 h, followed by the determination of the glycolysis rate using the glucose, lactic acid and ATP detection kit (Mean ± SD, n = 3). (J) Values are presented as the mean ± SD of three in Quantitative real‐time PCR analysis of mRNA expression of LDHB, LDHA, HK2, ENO1 and GAPDH treated with ATPR (10^−6^M) for 48h in MOLM‐13 and NB4 cells‐dependent experiments (K) The OCR was determined in MOLM‐13 and NB4 cells by Seahorse XF. *P < 0.05, **P < 0.01 versus control group

To identify whether RARα signalling was functional in AML cells treated with ATPR, we analysed the expression of retinoic acid receptor (RARα, RARβ, RARγ) and PML‐RARα by Western blotting in both NB4 and MOLM‐13 cells. We found that RARα and RARβ levels were elevated, and PML‐RARα levels were reduced (Figure 2A). This change was maintained until 72 h after treatment. However, unlike RARα, the expression levels of RARγ were little changed by ATPR treatment (*P* > 0.05). The expression of bona fide CRABP2 targets such as CRABP2 itself, CYP26A1 and RARβ, which promoters contain a RARE element, was detected by qRT‐PCR. All targets to be induced in cells treated with ATPR confirming a transcriptional activation (Figure 2B). Interestingly, RARα activation could also be observed in MOLM‐13 cells without PML‐RARα (Figure 2C, D). These findings suggested that ATPR activated RARα signalling and degraded PML‐RARα protein.

### 3.5 ATPR showed antileukaemic effect with RARα dependent

To further define whether RARα mediated the antileukaemic effect of ATPR, NB4 and MOLM‐13 cells were treated with a RARα antagonist (Ro41‐5253) prior to the addition of ATPR. As shown in Figure 2E, F, flow cytometry results showed that G0/G1 phase arrest and differentiation induction, which response to ATPR, were fully abolished by Ro41‐5253. The expression of Cyclin D3, Cyclin A2, CDK4 and PU.1 was also reversion (Figure 2G). These results showed that the antileukaemic effects of ATPR, including inhibition of proliferation and promotion of differentiation in AML cells, are RARα dependent.

### 3.6 The glycolysis situation of AML cells

To profile the glycolysis situation of AML cells, cells were stimulated with the glycolytic inhibitor, 2‐DG. CCK‐8 showed that 2‐DG inhibited the viability of AML cell lines in a concentration‐dependent manner (72 h). NB4 was the most sensitive, while KG‐1a was less sensitive to 2‐DG (Figure 2H). To investigate the effect of ATPR on the glycolysis rate of AML cells, the levels of glucose consumption, lactate production and ATP generation were tested. Our results showed that ATPR inhibited the levels of glucose consumption, lactate production and ATP generation only in NB4 and MOLM‐13 cells (Figure 2I). qRT‐PCR results showed that the expression of LDHB, LDHA, HK2, ENO1 and GAPDH was reduced after ATPR treatment, except GAPDH in both NB4 and MOLM‐13 cells (Figure 2J). Furthermore, the oxygen consumption rate (OCR) of NB4 and MOLM‐13 cells after ATPR treatment was evaluated by Seahorse XF. The results showed that ATPR treatment significantly decreased the rate of glycolysis and glycolytic capacity of both NB4 and MOLM‐13 cells (Figure 2K). Taken together, these results suggested that ATPR might exert antileukaemic effects by mediating glycolysis.

### 3.7 Modulation of ATPR‐inhibited LDHB expression by RARα‐selective agonist

**Figure 3:**
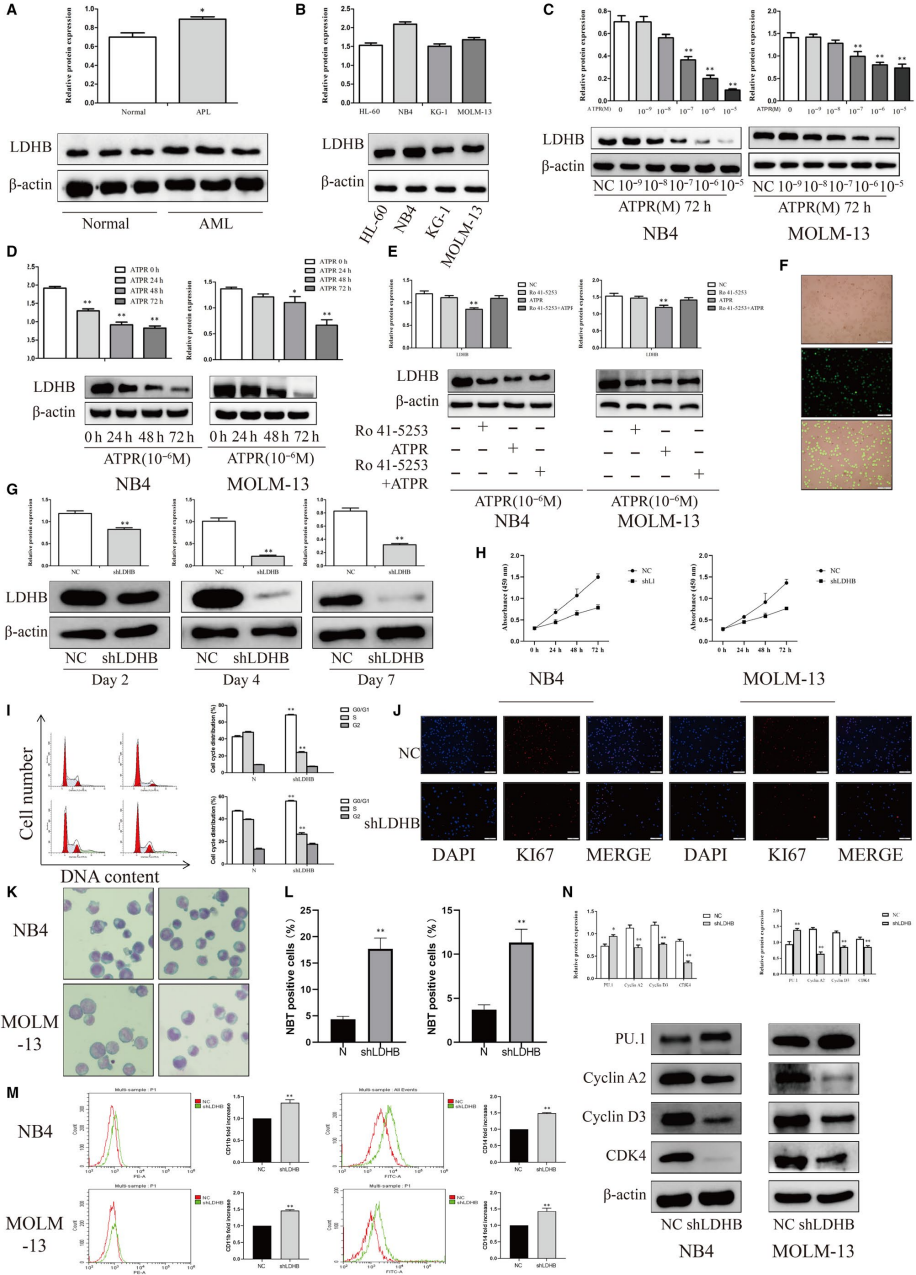
Knockdown LDHB expression inhibits proliferation while promoting differentiation and glycolysis of AML cells. (A) Western analysis of LDHB expression in AML patients PBMC compared with normal human PBMC (Mean ± SD, n = 3). (B) Western analysis of LDHB expression in AML cell lines (Mean ± SD, n = 3). (C, D) After treatment with ATPR (0, 10^−9^, 10^−8^, 10^−7^, 10^−6^, or 10^−5^ M) for different durations (24–72 h). The protein expression of LDHB was assessed by Western blotting (Mean ± SD, n = 3). (E) NB4 and MOLM‐13 cells were treated with ATPR in the absence or in the presence of the RARα‐selective antagonist Ro 41‐5253. The protein expression of LDHB was assessed by Western blotting (Mean ± SD, n = 3). (F) Stable control and shLDHB‐transfected NB4 cells were observed by inversed fluorescent microscope. (G) The protein expression of LDHB was assessed by Western blotting after treatment with shLDHB for 7 days (Mean ± SD, n = 3). (H) Cell growth of NB4 and MPOLM‐13 cells after transfection with shLDHB as determined by the CCK‐8 assay at different time points (Mean ± SD, n = 3). (I) The cell cycle distribution was analysed by flow cytometry in NB4 and MOLM‐13 cells after LDHB depletion (Mean ± SD, n = 3). (J) Immunofluorescence was used to detect KI67 in NB4 and MOLM‐13 cells after LDHB depletion. (K) After LDHB depletion, NB4 and MOLM‐13 cells were stained with Wright‐Giemsa dye and the cell morphological features were observed under a microscopy. (L) NBT reduction experiment was performed to count the positive cell rate (Mean ± SD, n = 3). (M) CD11b and CD14 expression were analysed by flow cytometer in NB4 and MOLM‐13 cells after LDHB depletion (Mean ± SD, n = 3). (N) The protein expression of PU.1, Cyclin A2, CDK4 and Cyclin D3 was assessed by Western blotting after LDHB depletion (Mean ± SD, n = 3). *P < 0.05, **P < 0.01 versus control group

To define the role of LDHB in AML, we analysed the expression of LDHB in normal human PBMC, AML patients PBMC and AML cell lines by Western blotting. We found that the expression of LDHB was increased in AML patients PBMC compared with normal human PBMC (Figure 3A). Furthermore, the expression of LDHB in NB4 cells was highest among different AML cell lines (Figure 3B). These indicated that the malignant phenotype of AML may be related to its glycolysis level.

To investigate whether the effect of ATPR on LDHB expression, we first examined the expression of LDHB in response to ATPR. In the present study, proteomics analysis identified 795 differentially expressed proteins after treatment with ATPR compared with control group. A total 6 down‐regulated proteins that had changes of < 0.677‐fold were found. Results of 6 down‐regulated proteins are displayed in Table 1. As shown in Figure 3C, D, after ATPR treatment, LDHB levels were decreased in a concentration‐ and time‐dependent manner.

**Table 1:** 8 down‐regulated proteins compared with control group

Description	Ratio
LDHB_HUMAN L_lactate dehydrogenase B chain OS = Homo sapiens GN = LDHB PE = 1 SV = 2	0.37
CALR_HUMAN Calreticulin OS = Homo sapiens GN = CALR PE = 1 SV = 1	0.39
HVCN1_HUMAN Voltage_gated hydrogen channel 1 OS = Homo sapiens GN = HVCN1 PE = 1 SV = 1	0.40
QCR2_HUMAN Cytochrome b_c1 complex subunit 2_ mitochondrial OS = Homo sapiens GN = UQCRC2 PE = 1 SV = 3	0.46
AT1A1_HUMAN Sodium/potassium_transporting ATPase subunit alpha_1 OS = Homo sapiens GN = ATP1A1 PE = 1 SV = 1	0.52
CHD4_HUMAN Isoform 2 of Chromodomain_helicase_DNA_binding protein 4 OS = Homo sapiens GN = CHD4	0.55
ILF3_HUMAN Isoform 7 of Interleukin enhancer_binding factor 3 OS = Homo sapiens GN = ILF3	0.57
RRBP1_HUMAN Ribosome_binding protein 1 OS = Homo sapiens GN = RRBP1 PE = 1 SV = 4	0.64

8 significantly down‐regulated proteins in the ATPR group compared with the control group. Values were presented as mean ± SD of three independent experiments. *P* < 0.05, ratio < 0.8

To confirm the direct involvement of the RARα signalling pathway in the modulation of ATPR‐inhibited LDHB expression, AML cells were treated with ATPR in the absence or in the presence of the RARα‐selective antagonist Ro 41‐5253. When AML cells were treated with Ro 41‐5253 prior to the addition of ATPR, the blockade of RARα signalling partially reduced the response of LDHB to ATPR (Figure 3E). These results confirmed that the expression of LDHB was regulated through the binding of ATPR to a RARα ligand‐binding domain.

### 3.8 Knockdown LDHB expression inhibited proliferation and glycolysis while promoting differentiation of AML cells

To confirm the above data derived from AML patients PBMC and to corroborate the function of LDHB in AML cells, we knocked down LDHB expression using shLDHB in NB4 and MOLM‐13 cells. Treatment with shLDHB significantly decreased the expression of LDHB compared with that in the control group (Figure 3F, G). The CCK‐8 assay results showed that the LDHB knockdown group inhibited cell proliferation more than the NC group (Figure 3H). Flow cytometry showed that the cell cycle was blocked at G0/G1 phase after shLDHB treatment (Figure 3I). KI‐67 dye showed that ATPR significantly decreased cell proliferation (Figure 3J). Wright‐Giemsa staining, NBT assay and flow cytometry analysis indicated that the level of cell differentiation was increased after shLDHB treatment (Figure 3K‐M). Furthermore, this conclusion was verified using Western blotting (Figure 3N). Collectively, these data revealed that LDHB potentially contributed to cell proliferation inhibition and differentiation induction in vitro.

### 3.9 ATPR regulated the Raf/MEK/ERK signalling pathway through LDHB

**Figure 4:**
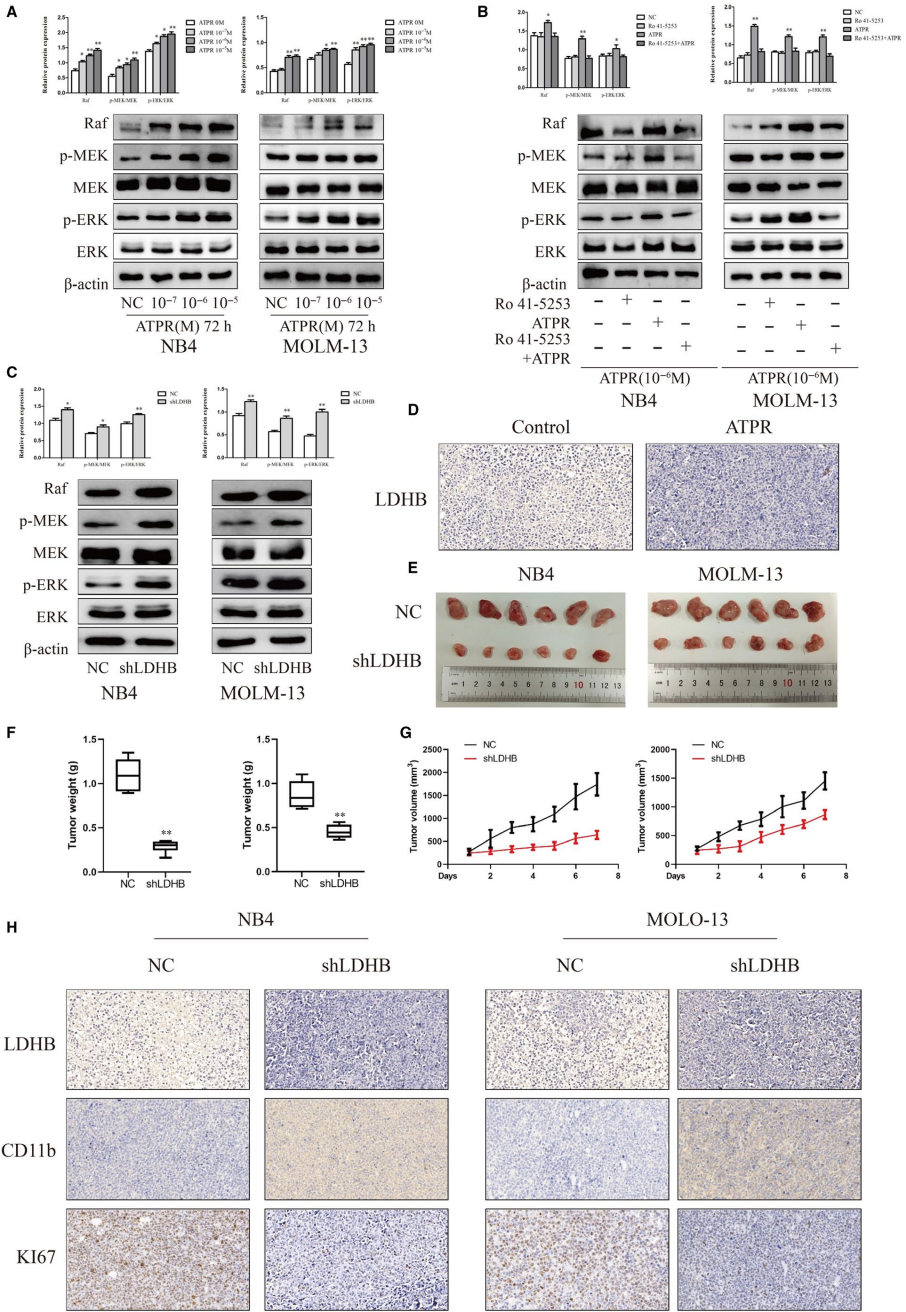
ATPR regulates the Raf/MEK/ERK signalling pathway through LDHB and the effects of LDHB on tumour growth in vivo (A) Protein expression of Raf, p‐MEK, MEK, p‐ERK and ERK was determined by Western blotting analysis after treatment with ATPR for different concentration (10^−7^,10^−6^,10^−5^) (Mean ± SD, n = 3). (B) Protein expression of Raf, p‐MEK, MEK, p‐ERK and ERK was determined by western blotting analysis after treatment with ATPR in the absence or in the presence of the RARα‐selective antagonist Ro 41‐5253 (Mean ± SD, n = 3). (C) Protein expression of Raf, p‐MEK, MEK, p‐ERK and ERK was determined by Western blotting analysis after LDHB depletion in NB4 and MOLM‐13 cells (Mean ± SD, n = 3). (D) Representative two tumour tissues from vehicle control mice and ATPR‐treated mice group was fixed and immunohistochemistry staining for LDHB. (E, F) Tumour images and weights at experimental endpoints in NC and shLDHB xenografts (Mean ± SD, n = 6). (G) Tumour volumes were measured every day (Mean ± SD, n = 6). (H) Immunohistochemistry staining for LDHB, CD11b and KI67 of NC and shLDHB in NB4 and MOLM‐13 group. *P < 0.05, **P < 0.01 versus control group

To test which signal pathway mediated the AML cells proliferation and differentiation in response to ATPR, the expression levels of proteins in the Raf/MEK/ERK pathway were detected. The results showed that the protein expression levels of Raf and phosphorylation levels of MEK and ERK proteins were significantly increased after ATPR treatment in a concentration‐dependent manner (Figure 4A).

To assess the importance of RARα in Raf/MEK/ERK pathway, AML cells were pretreated with Ro 41‐5253. Pharmacological inhibition of RARα blocked ATRA‐induced increases in Raf and phosphorylation levels of MEK and ERK protein expression (Figure 4B). Thus, our results demonstrated that RARα was required for activating Raf/MEK/ERK pathway in ATPR‐treated AML cells.

To explore whether the effect of LDHB on the Raf/MEK/ERK pathway, protein levels of Raf and phosphorylation levels of MEK and ERK were analysed in AML cells transfected with shLDHB and control. The results showed that the protein expression levels of Raf and phosphorylation levels of MEK and ERK proteins were significantly increased after transfection with shLDHB (Figure 4C). Taken together, these data indicated that ATPR regulated the Raf/MEK/ERK signalling pathway through LDHB.

### 3.10 Effects of LDHB on tumour growth in vivo

Our previous study had successfully constructed subcutaneous tumour in NCG mice. Compared with vehicle group, ATPR group inhibited the expression of LDHB in vivo (Figure 4D). In order to confirm the effect of LDHB on tumour formation in vivo, we first established a stable NB4 and MOLM‐13 cell lines using shLDHB. NCG mice received subcutaneous injections of shLDHB‐treated or control cells to establish the tumour model. After 2 weeks, tumours were completely stripped. Photographs and measured volumes of the tumours indicated that LDHB‐depleted cells grew much more slowly than control cells (Figure 4E, F). Moreover, the weights of the tumours from these mice were lower than those from control mice (Figure 4G). Immunohistochemical staining results indicated that CD11b was elevated and LDHB and KI67 were reduced (Figure 11H) . Together, these data demonstrated that LDHB knockdown inhibited tumour growth and induced differentiation in vivo by blocking the Raf/MEK/ERK signalling pathway in AML.

## 4 DISCUSSION

ATPR, a derivative of ATRA, designed and synthesized by the College of Pharmacy, Anhui Medical University. Our previous studies revealed that ATPR could exert the superior anticancer effects compared with ATRA in various cancers. However, the molecular mechanism by which ATPR affects cell differentiation and proliferation remains to be elucidated. In the present study, we explored the role of LDHB on ATPR‐induced differentiation and G0/G1 phase arrest in AML cells and determined the underlying mechanism. We first showed that pharmacological inhibition of RARα could block ATPR‐induced differentiation and G0/G1 phase, and this process was regulated by LDHB via Raf/MEK/ERK signalling pathway.

AML, caused by the abnormal proliferation of immature myeloid cells in the blood or bone marrow, is one of the most common haematologic malignancies.^20^ Accruing evidence indicates that ATRA is essential for AML treatment. The biological activity of ATRA is mediated by specific retinoic acid receptors (RARs), which act as ligand‐dependent transcription factors under the form of heterodimers with other retinoid receptors known as retinoid X receptors (RXRs). The RARs and RXRs form heterodimer and bind with RA response elements (RAREs) on the promoter of RA‐target gene activity and other protein complexes. After ATRA binding, HDAC activities are released from the RAR‐RXR heterodimer, and then transcriptional coactivators with intrinsic histone acetyltransferase activity are recruited into RARE ^21^. It has been confirmed that RARα is a transcription factor activated by ATRA and some RARα targets regulate myeloid differentiation and granulopoiesis ^22^, ^23^, ^24^. Therefore, we speculated that ATPR might inhibit cell proliferation and induce cell differentiation through RARα signalling like ATRA. As AML cells were exposed to Ro41‐5253, an effect of ATPR‐mediated growth inhibition was attenuated, and ATPR‐induced cell differentiation action was blocked. These results indicated that RARα was required for ATPR‐induced differentiation and proliferation inhibition in AML cells.

LDHB is a glycolytic enzyme that catalyses the conversion of lactate and NAD + to pyruvate, NADH and H+. The abundance of LDHB is abnormally increased in multiple types of cancer, including medulloblastoma, cholangiocarcinoma, oesophageal squamous cell carcinoma and breast cancer.^25^ Inhibitors targeting LDHB are likely to inhibit the growth of tumour cells. In this study, we found that LDHB was aberrantly overexpressed in human AML peripheral blood mononuclear cell (PBMC) and AML cell lines. Moreover, the expression of LDHB was the most significantly down‐regulated protein after ATPR treatment. Depletion of LDHB inhibited proliferation while promoting differentiation of AML in vitro and vivo. Additionally, LDHB expression was inversed correlated with Raf/MEK/ERK signalling pathway. Thus, it was reasonable to believe that knockdown LDHB could inhibit tumour growth and induce differentiation, which were essential for ATPR‐treated AML.

The typical MAPK signalling starts from the activation of receptor tyrosine kinase on the cell membrane and spreads through Raf/MEK/ERK in various biological processes, such as cell proliferation, cycle regulation, differentiation, survival and apoptosis.^26^, ^27^ It is well documented that multiple components of this signalling pathway provide attractive therapeutic targets in promoting disease progression and metastasis.^28^ However, the lack of success with MAPK components highlights the need for new treatment strategies. We and others have shown that ATPR could also crosstalk with this MAPK signalling to rapidly stimulate or suppress ERK phosphorylation in various cellular contexts.^29^, ^30^ We have also shown that ATPR could induce apoptosis via RARβ/RXRβ heterodimers and activation of ER stress involving the MAPK pathway in the breast cancer MDA‐MB‐231 cells.^31^ Furthermore, ATPR could induce cell differentiation in K562 cells, and its mechanism might be related to its ability in regulating the activation of ERK1/2 signalling pathway.^30^, ^32^ The present study also provided evidence for ATPR‐induced differentiation and proliferation inhibition in AML through regulating RARα/LDHB/ERK‐glycolysis signalling axis. We just investigated the effects of LDHB on differentiation and proliferation, and more studies are needed to research the potential molecular mechanism between LDHB and Raf/MEK/ERK pathway.

## 5 CONCLUSIONS

**Figure 5:**
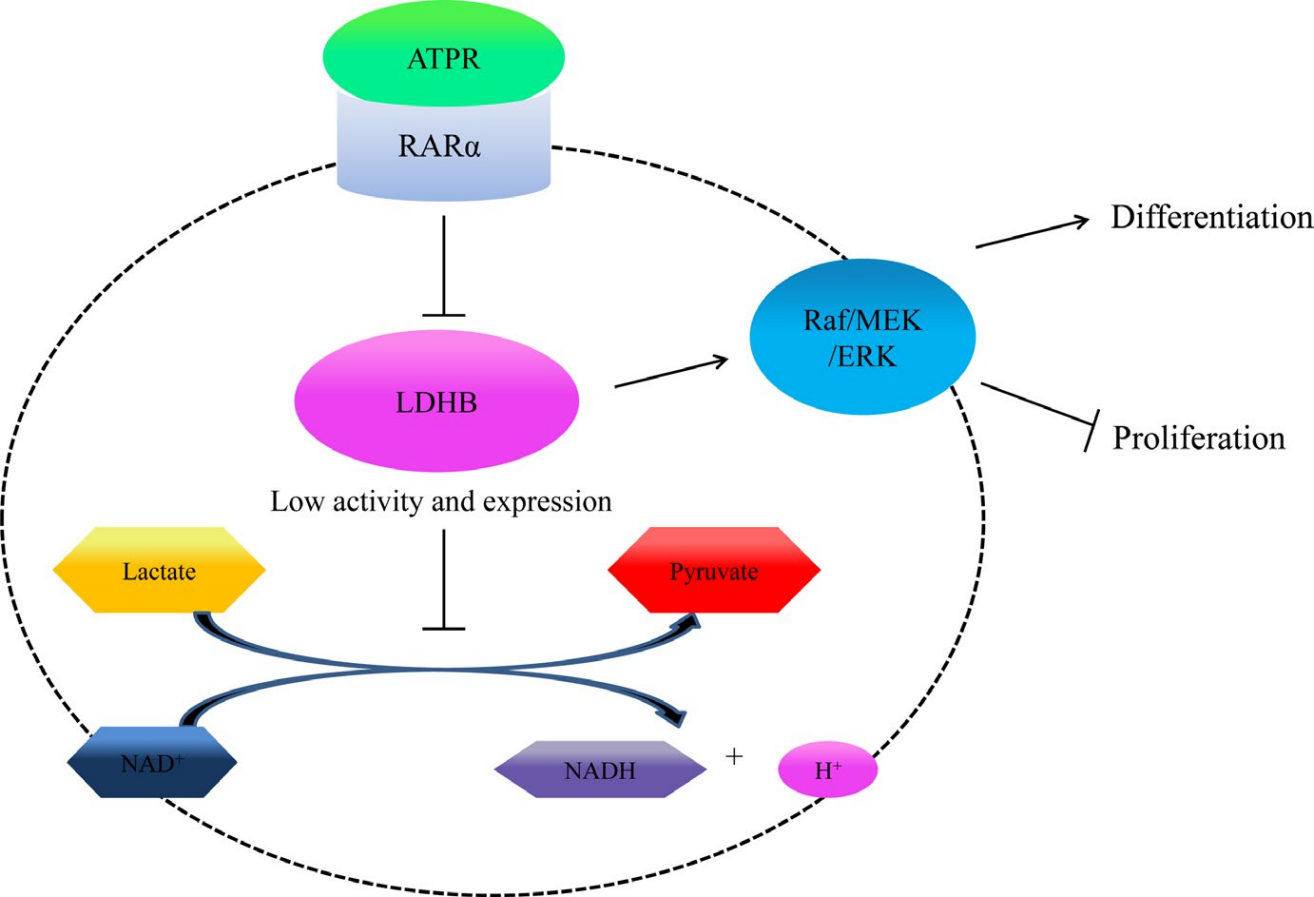
ATPR triggers AML cells differentiation and cycle arrest via the RARα/LDHB/ERK‐glycolysis signalling axis. ATPR triggers AML cells differentiation and cycle arrest via the RARα/LDHB/ERK‐glycolysis signalling axis

In summary, the present study suggests that inducing differentiation and inhibiting proliferation of ATPR is, at least partially, mediated by RARα/LDHB/ERK‐glycolysis signalling axis (Figure 5). Our finding highlights a novel mechanism underlying the effect of ATPR and discloses potential future therapeutic strategies for AML treatment.

## CONFLICT OF INTEREST

The authors declared no potential conflicts of interest concerning the research, authorship and publication of this article.

## AUTHORS’ CONTRIBUTIONS

Yan Du, Xiao‐qing Peng and Fei‐hu Chen participated in research design; Yan Du, Mei‐ju Zhang, Xiao‐Lin Xu, and Yu‐bin Feng conducted experiments; Fei‐hu Chen and Yan Li contributed new reagents or analytic tools; Lan‐lan Li and Hao Chen performed data analysis; Yan Du and Xiao‐qing Peng wrote or contributed to the writing of the manuscript.

## ETHICS APPROVAL AND CONSENT TO PARTICIPATE

The study was approved by written informed consent which was obtained from the parent or legal guardian according to the Declaration of Helsinki. All experiments involving live mice were approved by the Animal Care and Use Committee of Anhui Medical University (LLSC20190751).

## Data Availability

The data used to support the findings of this study are included within the article.
